# Enhancement of Lower Limb Muscle Strength and Reduction of Inflammation in the Elderly: A Randomized, Double-Blind Clinical Trial Comparing *Lacticaseibacillus paracasei* PS23 Probiotic with Heat-Treated Supplementation

**DOI:** 10.3390/nu17030463

**Published:** 2025-01-27

**Authors:** Mon-Chien Lee, Yi-Ju Hsu, Hung-Jen Yang, Chi-Chang Huang

**Affiliations:** 1 Graduate Institute of Sports Science, National Taiwan Sport University, Taoyuan City 333325, Taiwan; kurt0710@ntsu.edu.tw (M.-C.L.); ruby780202@ntsu.edu.tw (Y.-J.H.); 2 Center for General Education, Taipei Medical University, Taipei 110301, Taiwan; 3 Department of General Medicine, Min-Sheng General Hospital, Taoyuan City 330, Taiwan

**Keywords:** probiotics, postbiotics, *Lacticaseibacillus paracasei* PS23, sarcopenia, muscle strength

## Abstract

**Background**: As individuals age, there is a gradual loss of muscle mass and strength, which not only impairs physical functionality but also heightens the risk of falls and diminishes independence among older adults. Probiotics have emerged as a focus of recent research due to their potential role in enhancing muscle health via the gut–muscle axis. This study evaluates the effects of live and heat-treated *Lacticaseibacillus paracasei* PS23 (PS23) supplementation on muscle strength and mass in the elderly. **Methods:** This study recruited 119 participants, aged 65–85 years, and randomly assigned them to receive a placebo (0 × 10^10^ CFU/day), L-PS23 (live PS23, 2 × 10^10^ CFU/day), or HT-PS23 (heat-treated PS23, 2 × 10^10^ cells/day) for a duration of 12 weeks. Assessments of blood pressure, body composition, muscle strength, functional physical fitness, and biochemical parameters were conducted at baseline, 6 weeks, and 12 weeks. **Results:** Among the 100 subjects who completed the trial, supplementation with both L-PS23 and HT-PS23 significantly enhanced lower limb muscle strength and endurance compared to the placebo (*p* < 0.05), although no significant differences were observed in muscle mass or upper limb muscle strength across the groups. Additionally, while most muscle anabolism-related markers showed no significant changes, both supplements effectively decreased inflammatory markers related to aging—C-reactive protein (CRP: L-PS23, *p* = 0.016; HT-PS23, *p* = 0.013) and interleukin-6 (IL-6: L-PS23, *p* = 0.003; HT-PS23, *p* < 0.001)—and increased interleukin-10 levels (L-PS23, *p* = 0.014; HT-PS23, *p* = 0.005). Notably, only the HT-PS23 group demonstrated a significant increase in testosterone levels (*p* = 0.029). **Conclusions:** 12 weeks of supplementation with L-PS23 and HT-PS23 improved lower limb muscle strength and endurance but did not significantly enhance muscle mass in older adults. Both supplements also proved effective in reducing inflammatory markers and elevating testosterone levels. HT-PS23, administered as a heat-treated probiotic, provided more pronounced benefits to the elderly compared with its probiotic counterpart, L-PS23.

## 1. Introduction

The aging process entails numerous physiological changes, including a gradual loss of muscle mass and strength [1]. Muscle mass peaks around the age of 30 and subsequently declines by from approximately 3% to 8% each decade [2]. Beyond the age of 60, this decline accelerates, and, by the age of 70, muscle mass may decrease by from approximately 20% to 40%, leading to sarcopenia [3]. Sarcopenia is defined as a systemic skeletal muscle disorder characterized by a progressive decline in muscle mass and strength, which are recognized as critical parameters for its diagnosis. Specifically, a reduction in muscle mass is a key diagnostic criterion for sarcopenia, as outlined in the literature [4]. Furthermore, muscle strength begins to decline earlier and more precipitously than muscle mass. Starting at the age of 40, muscle strength declines annually by from 3% to 4% [5]. Sarcopenia, a systemic skeletal muscle disorder, often coexists with frailty [6] and is characterized by a progressive decline in homeostatic reserves, impairing the ability to maintain health and physical performance [7]. Contributing factors to this decline include inflammation, anabolic resistance, hormonal changes, alterations in the gut microbiota, DNA damage, increased oxidative stress, and mitochondrial dysfunction [8]. Consequently, frail elderly individuals may experience a reduction in muscle strength, gait speed, and endurance [9], which can lead to a loss of independence, increased risk of falls, fractures, hospitalizations, and even mortality. Moreover, these changes can exacerbate and lead to adverse physical conditions and diseases [10]. With advancements in global living standards and medical care, the average human lifespan has significantly increased, and the proportion of the global elderly population continues to rise [11]. Against this backdrop, it is crucial to develop effective aging strategies to promote a healthy lifespan, a challenge that requires urgent attention.

In recent years, the interplay between exercise, nutrition, and gut microbiota has garnered significant attention in the study of sarcopenia [12]. Notably, the gut microbiota has been implicated in regulating multiple muscle metabolic pathways [13]. Age-related changes in diet, lifestyle, and physiology contribute to a reduction in intestinal biodiversity and a decline in the number of species with health-promoting functions [14]. Previous research has demonstrated that variations in the relative abundance of gut microbiota are associated with muscle mass and physical frailty, with higher microbial diversity correlating with increased muscle mass [15]. As the concept of the ‘gut–muscle axis’ gains prominence, numerous studies have highlighted how derivatives and metabolites of gut microbiota can influence muscle functions [16]. These effects are mediated by mitigating inflammatory responses in the elderly, overcoming anabolic resistance, and enhancing skeletal muscle mass [16,17]. Although dysbiosis of the microbiota can lead to various diseases, the potential health benefits of modulating intestinal microbiota have increasingly become a focus of scientific inquiry [18].

Probiotic supplementation is considered one of the viable methods for regulating gut microbiota and enhancing gut homeostasis [19]. Animal studies have demonstrated that probiotics can influence muscle mass by increasing muscle weight, muscle fiber size, and the size of the tibialis muscle [20]. A meta-analysis that synthesized the findings from seven studies reported a positive effect of probiotic supplementation on muscle mass, although the supplementation period needs to extend beyond 12 weeks for significant benefits [21]. This effect is likely attributable to the ability of probiotics to reduce inflammation, enhance energy metabolism, and boost the synthesis of muscle hormones or factors [21,22,23]. Probiotics, primarily comprising Lactobacilli and Bifidobacteria, are defined as ‘live microorganisms which, when administered in adequate amounts, confer a health benefit on the host’ [24]. Moreover, probiotics can produce postbiotics through processes such as inactivation or metabolism, thus promoting health benefits for the host [25]. Despite being non-viable, these postbiotics—including inactivated microbial cells, cell wall components, functional proteins, peptides, short-chain fatty acids (SCFAs), bacteriocins, and other biologically active metabolites—still possess the capacity to inhibit the growth of pathogenic bacteria and enhance beneficial bacteria [26].

Previous research has demonstrated that *Lacticaseibacillus paracasei* PS23 can mitigate the decline in muscle mass and strength in aging mice by reducing inflammatory cytokines, reactive oxygen species, and enhancing mitochondrial performance, interleukin-10 (IL-10) levels, antioxidant enzyme activity, and protein uptake [27]. Heat-treated PS23 has also been shown to improve muscle strength and reduce muscle atrophy in aging mice by modulating ghrelin release [28]. A study involving elderly patients with sarcopenia revealed that a supplement containing leucine, omega-3 fatty acids, and probiotic *L. paracasei* PS23, administered over eight weeks, significantly enhanced lean body mass and grip strength [29]. However, the direct efficacy of PS23 alone in ameliorating muscle loss in older adults has not yet been evaluated. Consequently, this study aims to investigate the effects of both live and heat-treated *L. paracasei* PS23 administered over 12 consecutive weeks on the elderly. It will assess the improvement in muscle loss through measures of body composition, functional fitness testing, and the analysis of hormones, cytokines, and inflammatory factors in the blood related to muscle synthesis. Further, this research will compare the benefits and potential adverse effects of both probiotic and heat-treated forms of PS23 on the human body.

## 2. Materials and Methods

### 2.1. Subjects

The sample size calculation for this study was based on a power analysis to ensure sufficient statistical power to detect differences among the three groups. Using G*Power software (version 3.1.9.6), the parameters were set as follows: an assumed effect size (f) of 0.25, a type I error rate (α) of 0.05, and a power (1 − β) of 0.80. For a one-way ANOVA with three groups (placebo, live bacteria, heat-treated bacteria), the required total sample size was estimated to be 96 participants, with 32 participants per group. To account for potential dropouts, commonly reported at rates of 10–20% in clinical studies involving elderly populations, the total sample size by approximately 25% was increased, and, as a result, 119 participants in total were recruited. This adjustment ensures that the final analyzed sample size would remain sufficient to maintain this study’s statistical power.

**Figure 1:**
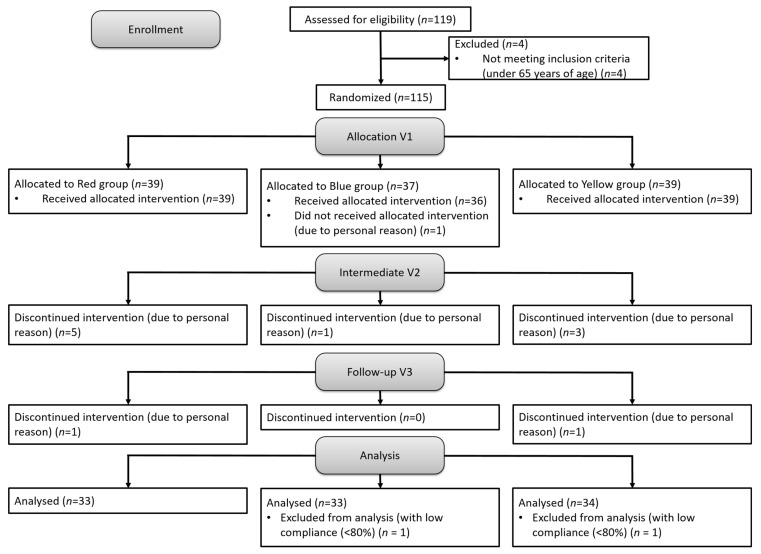
This figure outlines the step-by-step experimental procedures followed during this study.

This study recruited individuals aged 65–85 years. Participants were excluded if they met any of the following criteria: (1) recent use of antibiotics or receipt of antibiotic treatment within the past month; (2) recent use of probiotic products in powder, capsule, or lozenge form, with the exception of fermented milk products like yogurt and Yakult; (3) known allergies to lactic acid bacteria products; (4) presence of artificial joint replacements. A total of 119 participants met the inclusion criteria and were enrolled in this study. This study received ethical approval from the Institutional Review Board of Landseed International Hospital, Taoyuan, Taiwan (Approval No. LSHIRB 23-006-A2, approval date: 30 July 2023). All participants provided written informed consent before the commencement of this study. The trial was registered on clinicaltrials.gov (Identifier: NCT06062472) and was conducted in accordance with the Helsinki Declaration. Participants were randomly assigned to one of three groups: placebo (33 participants: 11 males, 22 females), live L-PS23 (33 participants: 12 males, 21 females), and heat-treated PS23 (HT-PS23; 34 participants: 16 males, 18 females). The experiment did not result in any adverse injuries. However, attrition occurred as some participants withdrew due to their inability to adhere to the experimental schedule or other personal factors. The experimental procedures, including interventions and assessments, are detailed in Figure 1. Throughout this study, data on demographic characteristics, compliance, and outcomes were collected and analyzed.

### 2.2. Experimental Design

All participants were enrolled in a double-blind, randomized controlled trial. Using their registration numbers, individuals were randomly assigned to one of three groups: a placebo group receiving microcrystalline cellulose (MCC, two capsules/day), a live *L. paracasei* PS23 group (L-PS23, 1 × 10^10^ CFU per capsule, two capsules/day), and a heat-treated *L. paracasei* PS23 group (HT-PS23, 1 × 10^10^ cells per capsule, two capsules/day). Bened Biomedical Co. Ltd. (Taipei, Taiwan) supplied the study capsules without any compensation. The dose setting was modified based on previous studies [27,29,30]. Participants were asked to take the corresponding supplement 30 min after breakfast every day for 12 weeks. Assessments were conducted at baseline, 6 weeks, and 12 weeks and included blood pressure measurements, body composition analysis, blood sample collection, functional fitness tests tailored for the elderly population, and muscle strength evaluations.

### 2.3. Blood Pressure and Body Composition Measurements

Participants were required to sit quietly in a chair for 5–10 min to ensure physical and mental relaxation prior to measurements. Blood pressure and heart rate were then assessed using an Omron HEM-1000 blood pressure monitor (Omron Healthcare, Taipei, Taiwan). This device recorded systolic blood pressure (SBP), diastolic blood pressure (DBP), and heart rate (HR).

Body composition was measured using the InBody 570 bioelectrical impedance analyzer (InBody, Seoul, Republic of Korea). This device employs a multi-frequency method, performing measurements at frequencies of 1, 5, 50, 260, 500, and 1000 kHz over a 60 s period. Participants were instructed to fast for 8 h prior to the measurement to ensure accuracy. During the measurement, participants were required to stand on the device’s sole electrodes, grasp the sensing handles with both hands, and maintain an open-arm posture angled at 30 degrees from the body. They were also advised to remain silent and motionless to prevent data variability, as previously described [30].

### 2.4. Handgrip Strength Test

Grip strength was quantified in kilograms using a Takei digital handgrip meter (T.K.K. 5401, Takei Scientific Instruments Co., Ltd., Kamo, Niigata, Japan). Prior to the formal testing, participants were instructed to apply minimal force to the gripper as a preliminary step to familiarize themselves with the operating procedures and the appropriate gripping distance. For the formal test, participants were asked to exert maximum effort by squeezing the gripper as forcefully as possible with one hand and maintaining the grip for at least five seconds. To mitigate the effects of fatigue, subjects alternated hands and repeated the test three times at 60 s intervals. The maximum grip strength for both the dominant and non-dominant hands was recorded [31].

### 2.5. Functional Performance

*Physical Fitness Test.* The physical fitness tests were conducted in accordance with established protocols and guidelines outlined in “The Fitness Guide for Older Adults”, published by the Sports Administration of the Ministry of Education [32,33]. The assessments included muscle strength, muscle endurance, flexibility, and balance, with each test’s methods and steps detailed below:

*30 s Arm Curl Test.* This test evaluates muscular endurance. Participants were seated with their dominant hand resting on the edge of a chair, feet flat on the floor, and maintaining an upright posture. They held a dumbbell in their dominant hand—3.63 kg for males and 2.27 kg for females—and performed arm curls. Starting with the arm fully extended, they curled the dumbbell towards the shoulder and then returned it to the starting position. The objective was to complete as many curls as possible within 30 s.

*10 m Walking Test.* Participants were required to walk a distance of 16 m as quickly as possible. Timing started 3 m beyond the starting point and stopped 3 m before the endpoint to prevent initial and final deceleration. The time taken to cover the central 10 m was recorded in seconds. The speed at which the central 10 m was covered provides an indication of lower limb endurance and walking ability.

*30 s Chair Stand Test.* This test assessed the muscle strength and endurance of the lower extremities. Participants sat in a chair placed against a wall, with feet shoulder-width apart and arms crossed at the wrists in front of the chest. On the “GO” command, they stood up and sat down as quickly as possible, repeating this action as many times as possible within 30 s. This test specifically measures lower limb strength and endurance, as the number of complete stands correlates directly with the strength and stamina of the lower extremities.

*2.44 m Timed Up-and-Go Test.* Designed to assess agility and dynamic balance, participants began seated in a chair with a triangular pyramid placed 2.44 m in front. Upon the start command, they stood up, navigated around the pyramid, and returned to sit in the chair. The time taken from standing up to sitting down was recorded, and the average of two trials was calculated. This test evaluates agility and dynamic balance, indirectly reflecting the overall function of lower limbs and muscle endurance, which are important for mobility in older adults.

*3 min Incremental Step-in-Place (3MISP) Test.* The 3MISP test was conducted to estimate maximal oxygen uptake (VO_2max_) [34]. Initially, the target height for knee elevation was established by measuring the midpoint between the patella and the iliac crest of each standing participant. This height was then marked with colored tape for consistency. Participants wore a Polar H10 Heart Rate Monitor (Polar Electro Oy, Kempele, Finland) equipped with a chest strap to continuously record heart rate (HR) during the test. The assessment commenced with participants stepping in place to the rhythm of an electronic metronome. The goal was to ensure that each knee lift met the pre-marked height. The starting step rate was set at 96 steps per minute (SPM), which increased by 24 SPM every minute up to a maximum duration of three minutes. Participants were allowed to shift from walking to running if necessary to maintain the rhythm. If a participant could not sustain the required knee height or rhythmic stepping for at least 30 s, the session was stopped, and their data were excluded from the analysis. To ensure safety, a 30 s cooldown period at a rate of 80 SPM was implemented immediately after the test. Heart rate measurements were taken at the start (HR0), during the first (HR1), second (HR2), and third minutes (HR3) of the exercise, as well as during the first minute post-exercise (HR4).

### 2.6. Clinical Biochemistry

*Sample Collection and Preparation*. At baseline, 6 weeks, and 12 weeks post-intervention, subjects were required to fast for at least 8 h prior to blood collection. Blood samples were drawn from the cubital vein by professional nursing staff. For the analysis of hemoglobin A1c (HbA1c), 1 mL of blood was collected into EDTA-containing tubes, while the remaining blood was centrifuged at 1500× *g* and 4 °C for 15 min to separate the serum.

*Biochemical Analysis*. Biochemical assays were conducted using the Beckman AU5800 automatic analyzer (Beckman Coulter Inc., Brea, CA, USA) to measure liver function, kidney function, blood lipids, and other biomarkers. Analyzed parameters included aspartate aminotransferase (AST), alanine transaminase (ALT), blood urea nitrogen (BUN), creatinine (CREA), uric acid (UA), glucose, calcium, total cholesterol (TC), triacylglycerol (TG), high-density lipoprotein (HDL), low-density lipoprotein (LDL), and high-sensitivity C-reactive protein (hs-CRP). Testosterone levels were measured using the Beckman DXI800 analyzer, and cystatin C was quantified with the Hitachi 7180 analyzer (Hitachi, Tokyo, Japan). Cortisol, insulin, and 25-hydroxyvitamin D were assessed using the Roche cobas e801 system (La Roche AG, Basel, Switzerland).

*Enzyme-Linked Immunosorbent Assay (ELISA)*. Additional markers were quantified using commercial ELISA kits. These included interleukin-6 (IL-6, D6050B), interleukin-10 (IL-10, D1000B), tumor necrosis factor-alpha (TNF-α, DTA00D), insulin-like growth factor-binding protein 3 (IGFBP-3, DGB300), growth/differentiation factor 15 (GDF-15, SGD150) from R&D Systems (Minneapolis, MN, USA), myeloperoxidase (S01410) from Cayman Chemical Co. (Ann Arbor, MI, USA), and cathepsin D (ab119586) from Abcam (Cambridge, UK). The absorbance was read using a Tecan Sunrise absorbance reader (Männedorf, Switzerland). Additionally, insulin-like growth factor-1 (IGF-1, IFU-A15729–03), human growth hormone (HGH, IFU-IM1397–01), dehydroepiandrosterone sulfate (DHEA-S, IFU-DSL2700–01) from Beckman Coulter, and ghrelin (GHRT-89HK) from Merck & Co. were measured using the Wallace Wizard 1470 Gamma Counter (PerkinElmer, Waltham, MA, USA).

### 2.7. Statistical Analysis

All data are presented as mean ± standard deviation (SD). Statistical analyses were conducted using SPSS Statistics 25 (IBM Co., Armonk, NY, USA). Comparisons across multiple groups were performed using a one-way analysis of variance (ANOVA). Differences between pre- and post-intervention measurements were assessed using two-way repeated-measures ANOVA, with Bonferroni corrections applied for multiple comparisons. For non-parametric datasets, the Wilcoxon signed-rank test was utilized. A significance level of *p* < 0.05 was established for all statistical tests, indicating a statistically significant difference.

## 3. Results

### 3.1. The Basic Information of Subjects and Effects of PS23 Supplementation on Blood Pressure and Body Composition in the Elderly

At the baseline, a total of 100 participants completed this study; the mean age of the participants was 69.3 ± 3.0 years, and the mean height was 160.3 ± 7.0 cm for the placebo group, 69.8 ± 3.0 years, and the mean height was 159.6 ± 6.6 cm for the L-PS23 group, and 69.9 ± 3.3 years, and the mean height was 160.0 ± 8.0 cm for the HT-PS23 group. No significant differences were observed in age or height between the three groups at baseline. Regarding body composition, no significant changes were noted in weight, body mass index (BMI), muscle mass, or body fat percentage across all groups at each time point (Table 1).

As reported in Table 1, no significant differences were observed in systolic blood pressure (SBP), diastolic blood pressure (DBP), and heart rate (HR) among the groups at 0, 6, and 12 weeks. However, there was a significant time-dependent effect on DBP (*p* = 0.018).

### 3.2. Effects of PS23 Supplementation on Grip Strength and Upper Limb Endurance in the Elderly

**Figure 2:**
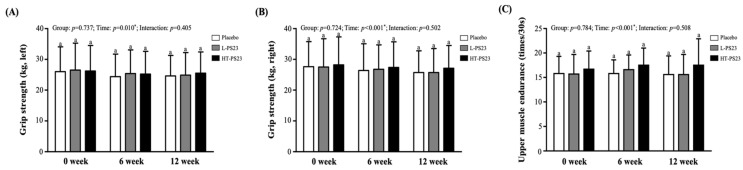
Effects of 12-week supplementation with L-PS23 and HT-PS23 on grip strength and Upper limb endurance in the elderly. (**A**) Left handgrip strength, (**B**) right handgrip strength, and (**C**) upper muscle endurance. Data are presented as mean ± standard deviation (SD). No significant differences were observed among groups at the same time points, as indicated by the same superscript letters (a), *p* > 0.05. An asterisk (*) indicates a significant effect as determined by two-way repeated-measures ANOVA with the Bonferroni post-hoc test (*p* < 0.05).

According to Figure 2, there were no significant differences in hand grip strength or upper limb muscle endurance among the placebo, L-PS23, and HT-PS23 groups throughout this study. Significant time effects were observed in left-hand grip strength (*p* = 0.010), right-hand grip strength (*p* < 0.001), and upper limb muscle endurance (*p* < 0.001).

### 3.3. Effects of PS23 Supplementation on Lower Limb Muscle Strength and Endurance in the Elderly

**Figure 3:**
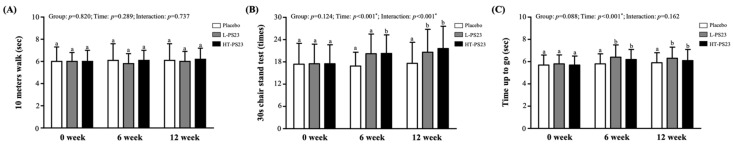
Effects of 12-week supplementation with L-PS23 and HT-PS23 on lower limb muscle strength and endurance in the elderly. (**A**) 10 m walk test, (**B**) 30 s chair stand test, and (**C**) timed up and go test. Data are presented as mean ± SD. Different superscript letters (a, b) indicate significant differences among groups at the same time point (*p* < 0.05). An asterisk (*) denotes a significant effect as determined by two-way repeated-measures ANOVA with the Bonferroni post-hoc test (*p* < 0.05).

The ability of the participants to perform lower limb strength and endurance was assessed using a 10 m walk (Figure 3A), 30 s chair stand tests (Figure 3B), and a 2.44 m timed up-and-go test (Figure 3C). There were no significant differences among groups in the 10 m walk test. However, the HT-PS23 group showed a significant 1.17-fold increase in the number of 30 s chair stands after 6 weeks (*p* = 0.031), and both the L-PS23 and HT-PS23 groups showed significant improvements after 12 weeks (*p* = 0.036 and *p* = 0.002, respectively). Similar trends were noted in the timed up-and-go test, with significant improvements noted in both L-PS23 and HT-PS23 groups at 6 weeks (*p* = 0.027 and *p* = 0.047, respectively) and at 12 weeks (*p* = 0.033 and *p* = 0.037, respectively).

### 3.4. Effect of PS23 Supplementation on Predicting Maximal Oxygen Uptake (VO_2max_) in the Elderly

**Figure 4:**
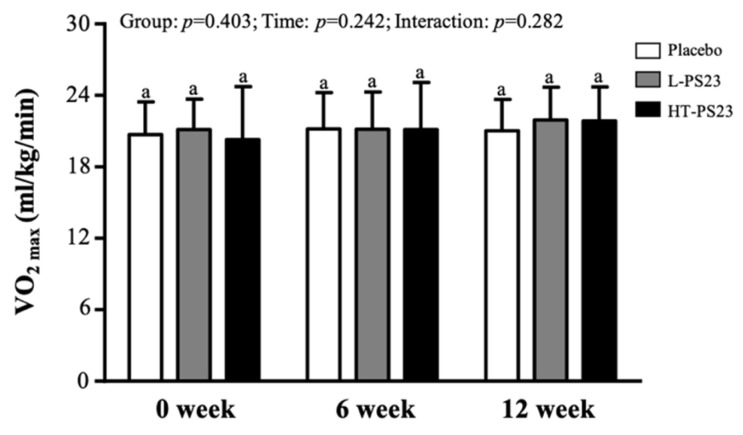
Effects of 12-week supplementation with L-PS23 and HT-PS23 on predicting maximal oxygen uptake (VO_2max_) in the elderly. Data are presented as mean ± SD. No significant differences were observed among groups at the same time points, as indicated by the same superscript letters (a), *p* > 0.05.

As depicted in Figure 4, supplementing with either L-PS23 or HT-PS23 for 12 consecutive weeks did not result in significant changes in VO_2max_.

### 3.5. Effects of PS23 Supplementation on Blood Indicators Related to Muscle Growth, Synthesis, or Inflammation in the Elderly

As detailed in Table 2, no significant differences were found among the groups for various markers including insulin, IGF-1, ghrelin, HsCRP, myeloperoxidase, GDF-15, TNF-α, cortisol, HGH, 25(OH)D, DHEA-s, cathepsin D, and cystatin C (CysC) after 12 weeks of supplementation. However, levels of IL-6 were significantly lower in both the L-PS23 and HT-PS23 groups compared to the placebo (*p* = 0.003 and *p* < 0.001, respectively), and IL-10 levels were significantly higher (*p* = 0.014 and *p* = 0.005, respectively). Notably, only the HT-PS23 group showed a significant increase in IGFBP-3 levels compared to the placebo group (*p* = 0.002). Testosterone levels were also significantly higher in the HT-PS23 group than in the placebo group after 6 and 12 weeks of supplementation (*p* = 0.035 each). Significant time effects were observed for several markers including IGF-1, IGFBP-3, ghrelin, IL-6, IL-10, TNF-α, testosterone, 25(OH)D, DHEA-s, and CysC. Furthermore, significant group × time interactions were noted for IGF-1, IGFBP-3, HsCRP, IL-6, IL-10, testosterone, and DHEA-s.

### 3.6. Effects of PS23 Supplementation on Biochemical Characteristics in the Elderly

Blood samples were analyzed at baseline and, subsequently, every 6 weeks following the initiation of the intervention to monitor basic blood parameters. These analyses helped assess the general health status of the participants and identify any potential adverse reactions or side effects associated with the 6-week PS23 supplementation regimen. The findings, detailed in Table 3, revealed no significant differences between groups in terms of liver function markers (aspartate aminotransferase [AST] and alanine transaminase [ALT]), renal function markers (blood urea nitrogen [BUN], creatinine [CREA], and uric acid [UA]), lipid profiles (total cholesterol [TC], triglycerides [TG], high-density lipoprotein cholesterol [HDL-C], and low-density lipoprotein cholesterol [LDL-C]), and glucose metabolism indicators (glucose and glycated hemoglobin [HbA1c]). Calcium (Ca) levels also remained unchanged before and after the intervention.

## 4. Discussion

The concept of the gut–muscle axis has garnered substantial interest, reflecting a growing body of research exploring the relationship between gut microbiota and muscle mass [35]. This interest has led to the strategic use of probiotics to potentially enhance muscle mass, especially among athletes and patients with sarcopenia [36]. However, most studies on age-related sarcopenia have relied predominantly on animal models, or have involved elderly subjects who are already experiencing sarcopenia, frailty, or other comorbid conditions [37]. Presently, investigations into the benefits of mitigating muscle loss among healthy older adults remain scarce. In the current study, we focused on this under-researched demographic by including older adults based solely on age criteria and administering L-PS23 or HT-PS23 continuously over 12 weeks. This study indicated that while there were no significant changes in overall muscle mass, there were notable enhancements in biomarkers linked to muscle synthesis, specifically IL-10, IGFBP-3, and testosterone. These results suggest that PS23 supplementation may contribute to an overall trend of improvement in muscle health, though these changes did not achieve statistical significance in terms of muscle mass. Significantly, the results demonstrate enhanced lower limb muscle strength and endurance performance. Noteworthy is that the heat-treated PS23 (HT-PS23) formulation appeared to confer greater benefits in these areas compared to the live L-PS23 version, suggesting potential advantages of postbiotic forms in this context (Table 4). This distinction between the effects of live and heat-treated formulations highlights the potential for tailored probiotic treatments depending on individual health status and specific therapeutic goals.

It is well-established that muscle mass loss accelerates with age, particularly in the absence of adequate physical activity or nutritional supplementation [38]. In this study, although no improvements in body composition were noted with PS23 supplementation, there was also no significant muscle loss observed in the placebo group as shown in Table 1. This outcome could be attributed to several factors. Firstly, recruitment was based solely on age without prior screening for sarcopenia or frailty. Additionally, the average age of participants may not have been sufficient to exhibit notable muscle loss, potentially leading to a “floor effect” [39]. Furthermore, participants were able to independently attend the experimental site and lived independently, suggesting that the extent of muscle loss among them might be less pronounced. It is also pertinent to mention that muscle strength declines more rapidly than muscle mass due to factors such as fatty infiltration of the muscles, neurological impairments, and reductions in muscle fiber number and size [40]. In addition to the findings presented in this study, it is important to consider the potential role of sex differences in muscle-wasting processes with aging. Previous research has shown that aging-related muscle loss, also known as sarcopenia, may manifest differently in males and females due to hormonal differences, variations in muscle fiber composition, and other factors. For instance, testosterone levels in men tend to decline with age, which can accelerate muscle loss, while women may experience a different pattern of muscle atrophy, often linked to hormonal changes such as menopause [41]. Importantly, loss of maximal strength is not uniform across all muscle groups, with muscle thickness, mass, and strength in the lower limbs being more vulnerable to age-related losses than those in the upper limbs [42]. This discrepancy is likely due to reduced engagement in activities like walking or running, whereas upper limb strength is maintained through routine daily activities [43]. Although evidence suggests that probiotics can enhance muscle strength performance in the elderly, comparisons between the effects on upper versus lower limb muscle strength remain unexplored. In this study, PS23 supplementation was found to significantly enhance muscle strength and endurance in the lower limbs of elderly participants, but it did not have a notable impact on upper limb strength or grip strength. This observation aligns with findings from a retrospective study that reported significant improvements in lower limb muscle strength following vitamin D supplementation, but not in the upper limbs [44]. Additionally, lower limbs typically bear more weight than upper limbs in daily activities and sports, which could further stimulate leg strength and enhance neuromuscular conditioning and capillary density [45]. This differential response suggests that lower limb muscle strength may be more receptive to interventions like vitamin D supplementation compared to upper limb muscles [44]. Nevertheless, further research is necessary to delineate whether probiotics exert disparate effects on upper and lower limb muscle strength, exploring the specific pathways and regulatory mechanisms involved.

In addition to assessments of muscle strength and functional performance, this study employed blood analysis to explore various physiological aspects such as metabolic regulation, inflammation, growth regulation, and cellular aging. This approach aimed to elucidate potential mechanisms by which L-PS23 and HT-PS23 influence muscle growth and breakdown. While most biochemical indicators showed no significant changes across all groups and remained consistent throughout the intervention period, a noteworthy finding was observed in the HT-PS23 group. After 12 consecutive weeks of supplementation, there was a significant increase in the blood concentration of IGFBP-3, as detailed in Table 2. IGF-1 and IGFBP-3 are pivotal in muscle development and maintenance [46]. IGF-1, a potent anabolic growth factor, stimulates the proliferation of muscle cells, contributing to an increase in muscle tissue mass and volume by enhancing protein synthesis within these cells [47]. IGFBP-3, primarily recognized as one of the main binding proteins of IGF-1, modulates the bioavailability and stability of IGF-1 [48]. The formation of an IGF-1/IGFBP-3 complex not only extends the half-life of IGF-1 in the bloodstream but also amplifies the growth-promoting effects of IGF-1 on muscle cells [49]. Importantly, IGFBP-3 also exerts direct regulatory effects on cell growth and differentiation independently of IGF-1, thereby contributing further to muscle growth [46]. Although the current literature does not directly demonstrate the effects of probiotics on IGFBP-3-mediated muscle synthesis, it is hypothesized that probiotic supplementation can enhance the composition and function of the gut microbiota, reduce inflammation and immune responses, and improve nutritional metabolism, potentially influencing IGFBP-3 levels [50]. However, more research is required to confirm these speculative effects and to explore the underlying mechanisms through which probiotics may influence muscle health.

Aging muscle is characterized by reduced mitochondrial volume and decreased oxidative capacity, leading to oxidative stress and subsequent inflammatory responses. This process is marked by the upregulation of inflammatory biomarkers such as C-reactive protein (CRP), IL-6, and TNF-α, all of which are associated with the development of sarcopenia [51,52]. Conversely, interleukin-10 (IL-10) is recognized as an anti-inflammatory cytokine, with decreases in IL-10 contributing to age-related inflammation [53]. Previous research has demonstrated that probiotic supplementation can mitigate inflammatory responses by enhancing the production of SCFA in the gut [54]. Additionally, probiotics have been shown to increase glutathione levels and reduce oxidative stress through the scavenging of superoxide and hydroxyl radicals, thereby decreasing IL-6 production in adipocytes [55]. Animal studies involving continuous administration of PS23 to aging mice for 12 weeks have shown significant enhancements in grip strength, protein digestibility, and mitochondrial function. These studies also reported reductions in age-related inflammation, including decreases in IL-6 levels and maintenance of IL-10 concentrations [27]. In line with animal studies, this study’s findings in human subjects suggest that both L-PS23 and HT-PS23 supplementation significantly reduce CRP and IL-6 levels while increasing IL-10 concentrations. These results support the potential of PS23 to regulate inflammation and mitigate muscle loss associated with aging. Moreover, probiotics may influence the metabolism and synthesis of hormones, including testosterone, which plays a crucial role in maintaining skeletal muscle quality and function [56]. Testosterone promotes bone development and protein synthesis within the musculoskeletal system, enhancing anabolism and acting directly on CD4+ cells to increase IL-10 expression [57,58]. In addition, a study involving Lactobacillus reuteri highlighted its potential to prevent age- and diet-related testicular atrophy in mice, suggesting broader applications for probiotics in hormone regulation [59]. Prior research indicated that PS23 supplementation could reduce testosterone decline and facilitate recovery from exercise-induced muscle damage in young adults [30]. Although the current study did not show significant changes in testosterone levels with L-PS23 or HT-PS23 supplementation, HT-PS23 notably increased testosterone concentrations in elderly participants, potentially offering a valuable intervention for sustaining muscle health with age. Testosterone and DHEA-S levels differ significantly between elderly men and women. Therefore, data for each sex were analyzed separately and are presented as Supplementary Data (Table S1).

Compared to L-PS23, HT-PS23 demonstrated more pronounced effects in reducing inflammatory markers (e.g., IL-6 and CRP) and increasing testosterone levels, as observed in this study. The enhanced stability and bioavailability of bioactive components in HT-PS23 due to heat treatment likely account for these differences. Heat treatment ensures that key bioactive components, such as cell wall components and metabolites, remain intact and active, even in the absence of viable cells [60]. This process minimizes variability in efficacy caused by the gradual degradation of live bacteria over time or under suboptimal storage conditions [61]. Additionally, the consistent bioactivity of HT-PS23 provides a practical advantage, particularly in clinical applications where storage, transportation, and handling conditions may compromise the viability of probiotics. While live bacteria in L-PS23 may also release bioactive components as they degrade, the predictability and stability of HT-PS23 make it a more reliable option for therapeutic use. These findings suggest that HT-PS23 could be particularly beneficial in populations where convenience and consistency are critical, such as elderly individuals with limited access to controlled storage environments.

Research on probiotic interventions specifically targeting the elderly remains sparse. Most human clinical trials focusing on aging explore aspects such as inflammation, infection, immune modulation, metabolic characteristics, cognitive function, gut microbiota, and quality of life [62]. Even fewer studies examine the effects of probiotics (live cells) and postbiotics (heat-inactivated cells) on muscle-related issues in the elderly using the same strains. The current study’s findings indicate that supplementation with L-PS23 significantly reduces inflammation and enhances lower limb muscle strength and endurance in the elderly. Remarkably, the heat-treated version, HT-PS23, conferred greater benefits than its live counterpart, aligning with results from aging animal models and exercise performance trials in younger individuals [28,30]. This enhanced efficacy could be attributed to the extended duration of our trial. Although we provided the test products to all participants every six weeks, the health benefits of probiotics are contingent on specific strains and dosages. These benefits can also be influenced by factors such as storage methods and the passage of time, which might lead to a gradual degradation or loss of live bacteria, thereby diminishing the total count of viable bacteria. In contrast, heat-treated postbiotics are less susceptible to degradation and offer numerous advantages, including increased safety, extended shelf life, and more straightforward transportation and storage [63]. Such characteristics make postbiotics particularly appealing in clinical settings, especially for elderly populations. A significant limitation of this study was the lack of observable improvements in muscle mass or the metabolic and hormonal indicators related to muscle synthesis. The mechanisms through which L-PS23 and HT-PS23 affect muscle synthesis and metabolism in the elderly remain unclear. Furthermore, several other factors should be considered. First, the relatively short duration of the 12-week intervention may not have been sufficient to fully capture the long-term effects of L-PS23 and HT-PS23 supplementation on muscle mass and metabolic markers. Longer studies could offer a clearer picture of the sustained benefits or potential delayed effects of these supplements on muscle health in older adults. Second, while the sample size was adequate for an initial investigation, a larger sample would improve statistical power and potentially yield more definitive results. A larger cohort would also help reduce variability and provide more reliable insights into the effects of the interventions across different subgroups, including gender and other demographic factors. Lastly, this study focused exclusively on elderly participants and did not include younger controls for comparison. Including younger subjects would have allowed for a better understanding of how age influences the effectiveness of probiotics and postbiotics in promoting muscle health, offering a broader context for interpreting the findings. Future research should aim to delineate these mechanisms more comprehensively. Additionally, exploring the roles of energy metabolism and gut microbiota in relation to both strains could provide valuable insights into more diversified therapeutic approaches.

## 5. Conclusions

This double-blind clinical trial investigated the effects of *L. paracasei* PS23 (L-PS23) and its heat-treated version (HT-PS23) on body composition and muscle strength in older adults. Although PS23 supplementation did not significantly enhance muscle mass, it did lead to substantial improvements in lower limb muscle strength and endurance performance over a 12-week period. While most metabolic and hormonal indicators related to muscle synthesis showed no significant changes, PS23 supplementation was effective in reducing inflammation markers associated with aging and significantly boosting testosterone levels. Notably, the heat-inactivated postbiotic form, HT-PS23, conferred greater benefits than the probiotic form, L-PS23. These findings suggest a promising role for both forms of PS23 in managing age-related muscle deterioration. However, further research is essential to fully understand the efficacy of PS23 supplementation, necessitating additional studies on optimal supplementation duration, dosage, and the underlying mechanisms.

## Figures and Tables

**Table 1:** Changes in subjects’ blood pressure and body composition following 6-week and 12-week interventions with L-PS23 and HT-PS23.

Characteristics	Time	Placebo	L-PS23	HT-PS23	Group (G)	Time (T)	G × T
Body composition							
BW (kg)	0 week	60.8 ± 10.5 ^a^	59.6 ± 9.9 ^a^	61.6 ± 10.1 ^a^	0.748	0.302	0.494
	6 week	60.6 ± 10.5 ^a^	59.6 ± 9.9 ^a^	61.6 ± 10.3 ^a^			
	12 week	60.8 ± 10.7 ^a^	59.8 ± 10.1 ^a^	61.6 ± 10.2 ^a^			
BMI (kg/m^2^)	0 week	23.6 ± 3.2 ^a^	23.3 ± 2.7 ^a^	24.0 ± 3.3 ^a^	0.471	0.235	0.493
	6 week	23.5 ± 3.3 ^a^	23.3 ± 2.7 ^a^	24.0 ± 3.3 ^a^			
	12 week	23.6 ± 3.3 ^a^	23.4 ± 2.8 ^a^	24.0 ± 3.3 ^a^			
Muscle mass (kg)	0 week	22.7 ± 5.0 ^a^	22.8 ± 4.3 ^a^	23.4 ± 4.5 ^a^	0.767	0.132	0.638
	6 week	22.7 ± 4.9 ^a^	23.0 ± 4.3 ^a^	23.5 ± 4.4 ^a^			
	12 week	22.7 ± 5.0 ^a^	23.0 ± 4.2 ^a^	23.5 ± 4.4 ^a^			
Fat mass (%)	0 week	30.9 ± 5.8 ^a^	28.8 ± 6.5 ^a^	29.5 ± 7.0 ^a^	0.482	0.115	0.379
	6 week	30.6 ± 5.7 ^a^	28.6 ± 6.2 ^a^	29.2 ± 6.8 ^a^			
	12 week	30.1 ± 6.0 ^a^	28.7 ± 6.4 ^a^	29.4 ± 6.7 ^a^			
Blood pressure							
SBP (mmHg)	0 week	134.1 ± 15.3 ^a^	140.2 ± 20.7 ^a^	133.9 ± 18.2 ^a^	0.582	0.062	0.637
	6 week	130.6 ± 14.6 ^a^	135.0 ± 19.5 ^a^	132.1 ± 19.9 ^a^			
	12 week	134.9 ± 17.5 ^a^	136.1 ± 19.1 ^a^	134.9 ± 23.4 ^a^			
DBP (mmHg)	0 week	78.5 ± 9.3 ^a^	76.9 ± 11.5 ^a^	76.4 ± 10.3 ^a^	0.722	0.018 *	0.911
	6 week	76.3 ± 8.2 ^a^	73.8 ± 13.8 ^a^	73.6 ± 10.3 ^a^			
	12 week	77.0 ± 11.0 ^a^	76.5 ± 11.8 ^a^	76.4 ± 14.1 ^a^			
HR (bpm)	0 week	76.2 ± 10.9 ^a^	75.4 ± 9.3 ^a^	74.2 ± 10.2 ^a^	0.486	0.675	0.397
	6 week	78.1 ± 14.1 ^a^	74.2 ± 10.2 ^a^	74.5 ± 10.3 ^a^			
	12 week	77.6 ± 10.9 ^a^	74.3 ± 11.0 ^a^	76.0 ± 11.1 ^a^			

Data are presented as mean ± standard deviation (SD). No significant differences were observed among groups at the same time points, as indicated by the same superscript letters (a), *p* > 0.05. Key abbreviations used include systolic blood pressure (SBP), diastolic blood pressure (DBP), HR (heart rate), BW (body weight) and BMI (body mass index), and group × time interaction (G × T). An asterisk (*) indicates a significant effect as determined by two-way repeated-measures ANOVA with the Bonferroni post-hoc test (*p* < 0.05).

**Table 2:** Effect of PS23 supplementation on various parameters.

Characteristics	Time	Placebo	L-PS23	HT-PS23	Group (G)	Time (T)	G × T
Insulin (μU/mL)	0 week	8.10 ± 3.16 ^a^	8.27 ± 3.63 ^a^	7.91 ± 3.84 ^a^	0.830	0.204	0.286
	6 week	7.46 ± 3.30 ^a^	8.00 ± 3.69 ^a^	7.76 ± 4.23 ^a^			
	12 week	7.13 ± 3.46 ^a^	8.02 ± 3.29 ^a^	8.13 ± 5.44 ^a^			
IGF-1 (ng/mL)	0 week	107.6 ± 39.2 ^a^	101.8 ± 46.5 ^a^	100.3 ± 34.1 ^a^	0.977	<0.001 *	0.031 *
	6 week	106.4 ± 36.6 ^a^	106.1 ± 47.3 ^a^	101.8 ± 30.4 ^a^			
	12 week	110.0 ± 33.2 ^a^	121.2 ± 56.1 ^a^	117.4 ± 38.3 ^a^			
IGFBP-3 (pg/mL)	0 week	2488 ± 495 ^a^	2366 ± 656 ^a^	2417 ± 673 ^a^	0.433	<0.001 *	0.002 *
	6 week	2425 ± 632 ^a^	2498 ± 722 ^a^	2550 ± 826 ^a^			
	12 week	2394 ± 497 ^a^	2666 ± 612 ^a^	2883 ± 756 ^b^			
Ghrelin (pmol/L)	0 week	922 ± 345 ^a^	928 ± 376 ^a^	927 ± 322 ^a^	0.514	0.009 *	0.344
	6 week	1053 ± 264 ^a^	995 ± 189 ^a^	982 ± 208 ^a^			
	12 week	1062 ± 330 ^a^	985 ± 291 ^a^	927 ± 209 ^a^			
HsCRP (mg/dL)	0 week	0.09 ± 0.07 ^a^	0.09 ± 0.07 ^a^	0.10 ± 0.08 ^a^	0.503	0.185	0.001 *
	6 week	0.09 ± 0.07 ^a^	0.09 ± 0.07 ^a^	0.10 ± 0.06 ^a^			
	12 week	0.13 ± 0.09 ^b^	0.09 ± 0.07 ^a^	0.09 ± 0.07 ^a^			
Myeloperoxidase (ng/mL)	0 week	255 ± 88 ^a^	246 ± 82 ^a^	254 ± 102 ^a^	0.606	0.994	0.622
	6 week	258 ± 95 ^a^	236 ± 85 ^a^	257 ± 107 ^a^			
	12 week	267 ± 91 ^a^	236 ± 88 ^a^	250 ± 93 ^a^			
GDF-15 (pg/mL)	0 week	895 ± 425 ^a^	960 ± 466 ^a^	1039 ± 557 ^a^	0.433	0.023	0.406
	6 week	843 ± 582 ^a^	866 ± 417 ^a^	999 ± 561 ^a^			
	12 week	909 ± 635 ^a^	880 ± 430 ^a^	1059 ± 631 ^a^			
IL-6 (pg/mL)	0 week	2.24 ± 0.88 ^a^	2.46 ± 0.77 ^a^	2.44 ± 0.80 ^a^	0.205	<0.001 *	<0.001 *
	6 week	2.24 ± 0.88 ^a^	2.18 ± 0.81 ^a^	2.11 ± 0.80 ^a^			
	12 week	2.41 ± 1.00 ^b^	1.77 ± 0.84 ^a^	1.47 ± 0.72 ^a^			
IL-10 (pg/mL)	0 week	0.70 ± 0.41 ^b^	0.48 ± 0.36 ^a^	0.46 ± 0.26 ^a^	0.905	0.048 *	<0.001 *
	6 week	0.56 ± 0.43 ^a^	0.46 ± 0.35 ^a^	0.41 ± 0.32 ^a^			
	12 week	0.39 ± 0.29 ^a^	0.64 ± 0.53 ^b^	0.68 ± 0.38 ^b^			
TNF-α (pg/mL)	0 week	0.20 ± 0.06 ^a^	0.19 ± 0.06 ^a^	0.21 ± 0.08 ^a^	0.592	<0.001 *	0.370
	6 week	0.24 ± 0.07 ^a^	0.23 ± 0.06 ^a^	0.24 ± 0.07 ^a^			
	12 week	0.24 ± 0.08 ^a^	0.22 ± 0.07 ^a^	0.22 ± 0.07 ^a^			
Testosterone (ng/mL)	0 week	1.70 ± 1.26 ^a^	1.60 ± 0.84 ^a^	1.72 ± 1.26 ^a^	0.221	0.001 *	<0.001 *
	6 week	1.75 ± 1.25 ^a^	1.56 ± 0.88 ^a^	2.19 ± 1.44 ^b^			
	12 week	1.60 ± 1.20 ^a^	1.62 ± 0.91 ^a^	2.29 ± 1.58 ^b^			
Cortisol (μg/dL)	0 week	8.82 ± 2.65 ^a^	9.53 ± 3.00 ^a^	10.02 ± 2.98 ^b^	0.544	0.082	0.599
	6 week	8.83 ± 3.05 ^a^	9.03 ± 2.82 ^a^	9.64 ± 4.20 ^a^			
	12 week	8.72 ± 2.94 ^a^	8.88 ± 2.94 ^a^	8.86 ± 3.14 ^a^			
HGH (ng/mL)	0 week	1.02 ± 0.59 ^a^	0.89 ± 0.46 ^a^	0.99 ± 0.61 ^a^	0.863	0.968	0.313
	6 week	1.03 ± 0.60 ^a^	0.93 ± 0.46 ^a^	0.97 ± 0.55 ^a^			
	12 week	0.88 ± 0.56 ^a^	0.98 ± 0.48 ^a^	1.03 ± 0.69 ^a^			
25(OH)D (ng/mL)	0 week	24.4 ± 4.7 ^a^	26.4 ± 8.2 ^a^	26.3 ± 8.0 ^a^	0.331	<0.001 *	0.688
	6 week	25.4 ± 4.8 ^a^	28.5 ± 8.3 ^a^	27.9 ± 7.6 ^a^			
	12 week	24.4 ± 4.9 ^a^	26.4 ± 8.7 ^a^	26.2 ± 8.6 ^a^			
DHE^A^-S (μg/dL)	0 week	103.6 ± 69.6 ^a^	113.3 ± 50.5 ^a^	106.0 ± 47.6 ^a^	0.519	<0.001 *	0.007 *
	6 week	109.7 ± 61.1 ^a^	125.4 ± 57.5 ^a^	116.2 ± 51.7 ^a^			
	12 week	118.4 ± 65.3 ^a^	140.6 ± 70.2 ^a^	142.5 ± 56.8 ^a^			
Cathepsin D (ng/mL)	0 week	24.5 ± 6.5 ^a^	24.7 ± 10.1 ^a^	25.0 ± 8.6 ^a^	0.425	0.198	0.125
	6 week	24.4 ± 6.3 ^a^	26.4 ± 9.1 ^a^	26.8 ± 7.7 ^a^			
	12 week	22.7 ± 6.7 ^a^	26.7 ± 9.2 ^a^	26.4 ± 9.6 ^a^			
CysC (mg/L)	0 week	0.88 ± 0.15 ^a^	0.89 ± 0.13 ^a^	0.91 ± 0.19 ^a^	0.355	0.005 *	0.529
	6 week	0.84 ± 0.13 ^a^	0.86 ± 0.15 ^a^	0.91 ± 0.24 ^a^			
	12 week	0.82 ± 0.15 ^a^	0.86 ± 0.18 ^a^	0.90 ± 0.24 ^a^			

Data are presented as mean ± SD. Different superscript letters (a, b) denote significant differences among groups at the same time point, *p* < 0.05. An asterisk (*) indicates a significant effect as determined by two-way repeated-measures ANOVA with the Bonferroni post-hoc test (*p* < 0.05). Abbreviations used are as follows: IGF-1 (insulin-like growth factor-1), IGFBP-3 (insulin-like growth factor-binding protein 3), HsCRP (high sensitivity C-reactive protein), GDF-15 (growth/differentiation factor 15), IL-6 (interleukin-6), TNF-α (tumor necrosis factor-alpha), HGH (human growth hormone), DHEA-S (dehydroepiandrosterone sulfate), and CysC (cystatin C).

**Table 3:** Effects of L-PS23 and HT-PS23 supplementation on biochemical characteristics in the elderly.

Characteristics	Time	Placebo	L-PS23	HT-PS23	Group (G)	Time (T)	G × T
AST (U/L)	0 week	22 ± 5 ^a^	22 ± 4 ^a^	20 ± 3 ^a^	0.199	0.047 *	0.686
	6 week	21 ± 5 ^a^	21 ± 5 ^a^	20 ± 4 ^a^			
	12 week	21 ± 6 ^a^	22 ± 5 ^a^	20 ± 4 ^a^			
ALT (U/L)	0 week	18 ± 7 ^a^	17 ± 7 ^a^	17 ± 5 ^a^	0.594	0.014 *	0.858
	6 week	17 ± 6 ^a^	16 ± 6 ^a^	16 ± 4 ^a^			
	12 week	17 ± 8 ^a^	16 ± 6 ^a^	16 ± 4 ^a^			
BUN (mg/dL)	0 week	16.6 ± 3.0 ^a^	16.7 ± 4.5 ^a^	16.6 ± 4.3 ^a^	0.594	0.074	0.858
	6 week	16.7 ± 3.7 ^a^	16.3 ± 3.7 ^a^	16.6 ± 4.1 ^a^			
	12 week	16.3 ± 3.4 ^a^	16.3 ± 3.7 ^a^	16.6 ± 4.1 ^a^			
CREA (mg/dL)	0 week	0.80 ± 0.15 ^a^	0.81 ± 0.23 ^a^	0.81 ± 0.17 ^a^	0.984	0.057	0.758
	6 week	0.79 ± 0.15 ^a^	0.79 ± 0.23 ^a^	0.79 ± 0.18 ^a^			
	12 week	0.79 ± 0.16 ^a^	0.78 ± 0.24 ^a^	0.80 ± 0.16 ^a^			
UA (mg/dL)	0 week	5.1 ± 1.4 ^a^	5.2 ± 1.0 ^a^	5.2 ± 1.0 ^a^	0.623	0.115	0.491
	6 week	4.9 ± 1.4 ^a^	5.0 ± 1.0 ^a^	5.2 ± 0.9 ^a^			
	12 week	4.9 ± 1.3 ^a^	4.9 ± 0.9 ^a^	5.3 ± 1.1 ^a^			
Glucose (mg/dL)	0 week	88 ± 18 ^a^	88 ± 12 ^a^	88 ± 16 ^a^	0.998	0.780	0.981
	6 week	89 ± 20 ^a^	89 ± 12 ^a^	89 ± 23 ^a^			
	12 week	89 ± 17 ^a^	88 ± 12 ^a^	88 ± 13 ^a^			
HbA1c (%)	0 week	5.8 ± 0.6 ^a^	5.8 ± 0.4 ^a^	5.9 ± 0.5 ^a^	0.870	0.054	0.921
	6 week	5.8 ± 0.7 ^a^	5.8 ± 0.4 ^a^	5.9 ± 0.5 ^a^			
	12 week	5.8 ± 0.6 ^a^	5.9 ± 0.4 ^a^	5.9 ± 0.5 ^a^			
TG (mg/dL)	0 week	110 ± 40 ^a^	112 ± 49 ^a^	107 ± 33 ^a^	0.912	0.177	0.942
	6 week	108 ± 39 ^a^	112 ± 56 ^a^	106 ± 46 ^a^			
	12 week	106 ± 36 ^a^	105 ± 48 ^a^	104 ± 40 ^a^			
TC (mg/dL)	0 week	189 ± 31 ^a^	182 ± 23 ^a^	190 ± 33 ^a^	0.588	0.068	0.823
	6 week	186 ± 30 ^a^	178 ± 24 ^a^	183 ± 34 ^a^			
	12 week	182 ± 38 ^a^	178 ± 28 ^a^	185 ± 41 ^a^			
HDL (mg/dL)	0 week	59.3 ± 12.9 ^a^	58.9 ± 11.8 ^a^	58.7 ± 10.4 ^a^	0.972	0.302	0.394
	6 week	58.4 ± 13.7 ^a^	58.4 ± 11.5 ^a^	56.7 ± 11.8 ^a^			
	12 week	58.6 ± 15.6 ^a^	57.3 ± 12.2 ^a^	59.2 ± 14.4 ^a^			
LDL (mg/dL)	0 week	113.3 ± 26.1 ^a^	106.8 ± 22.5 ^a^	114.0 ± 29.7 ^a^	0.611	0.004 *	0.682
	6 week	110.5 ± 28.8 ^a^	103.4 ± 22.4 ^a^	108.4 ± 27.5 ^a^			
	12 week	105.7 ± 28.6 ^a^	103.5 ± 24.1 ^a^	107.5 ± 32.4 ^a^			
Ca (mg/dL)	0 week	9.3 ± 0.6 ^a^	9.2 ± 0.6 ^a^	9.2 ± 0.5 ^a^	0.872	0.128	0.358
	6 week	9.1 ± 0.6 ^a^	9.0 ± 0.5 ^a^	9.0 ± 0.6 ^a^			
	12 week	9.0 ± 0.8 ^a^	9.1 ± 0.6 ^a^	9.2 ± 0.6 ^a^			

Data are presented as mean ± SD. The same superscript letters (a) across groups at the same time point indicate no significant difference, *p* > 0.05. An asterisk (*) denotes a significant effect determined by two-way repeated-measures ANOVA with the Bonferroni post-hoc test, *p* < 0.05. Abbreviations: AST, aspartate aminotransferase; ALT, alanine aminotransferase; BUN, blood urea nitrogen; CREA, creatine; UA, uric acid; HbA1c, glycated Hemoglobin; TC, total cholesterol; TG, triacylglycerol; HDL, high-density lipoprotein; LDL, low-density lipoprotein.

**Table 4:** Summarizes the key differences in the actions of L-PS23 and HT-PS23.

Items	L-PS23	HT-PS23
Enhancement of lower limb muscle strength	Yes	Yes (greater improvement compared to L-PS23)
Reduction of inflammatory markers (e.g., IL-6, CRP)	Yes	Yes (more pronounced effect on IL-6 and CRP reduction)
Increase in anti-inflammatory marker (IL-10)	Yes	Yes (slightly higher increase compared to L-PS23)
Increase in testosterone levels	No significant effect	Yes (significant increase observed)
Effects on muscle mass	No significant change	No significant change
Stability and ease of storage	Limited by viability of live cells	High (due to heat inactivation)

## Data Availability

Data is contained within the article.

## Supplementary Materials

The following supporting information can be downloaded at www.mdpi.com/article/10.3390/nu17030463/s1: Table S1. Effects of PS23 Supplementation on Testosterone and DHEA-S in Different Genders.
