# Supplementary material for: Enhancement of Lower Limb Muscle Strength and Reduction of Inflammation in the Elderly: A Randomized, Double-Blind Clinical Trial Comparing Lacticaseibacillus paracasei PS23 Probiotic with Heat-Treated Supplementation
Source: Nutrients. 2025 Jan 27;17(3):463. doi: 10.3390/nu17030463 (PMC11820367; doi:10.3390/nu17030463)
Supplement: Supplementary file 1 [file nutrients-17-00463-s001.zip › nutrients-3439194-supplementary.pdf]

Table S1. Effects of PS23 Supplementation on Testosterone and DHEA-S in Different Genders.

Characteristics	Genders	Group	Placebo	L-PS23	HT-PS23	Group (G)	Time (T)	G×T
**Testosterone (ng/mL)**	**Male**	0 week	3.16±0.87^{a}	2.49±0.32^{a}	2.60±1.08^{a}	0.016*	0.128	<0.001*
		6 week	3.24±0.71^{b}	2.26±0.59^{a}	3.19±0.70^{b}			
		12 week	2.97±0.80^{ab}	2.39±0.52^{a}	3.46±0.83^{b}			
	**Female**	0 week	0.98±0.63^{a}	1.09±0.57^{a}	0.94±0.83^{a}	0.706	0.010*	0.030
		6 week	1.01±0.64^{a}	1.16±0.76^{a}	1.31±1.35^{a}			
		12 week	0.92±0.64^{a}	1.19±0.78^{a}	1.24±0.34^{a}			
**DHEA-S (ug/dL)**	**Male**	0 week	142.8±92.8^{a}	123.9±60.4^{a}	137.2±35.7^{a}	0.892	<0.001*	0.034*
		6 week	137.4±79.6^{a}	146.6±69.9^{a}	145.2±42.3^{a}			
		12 week	149.9±81.0^{a}	163.8±87.7^{a}	179.5±47.9^{a}			
	**Female**	0 week	84.0±45.6^{a}	107.2±44.3^{a}	78.2±39.3^{a}	<0.001*	<0.001*	<0.001*
		6 week	95.8±45.5^{a}	113.4±46.7^{a}	90.4±46.0^{a}			
		12 week	102.6±50.9^{a}	127.3±56.2^{a}	109.6±42.5^{a}			

Data are presented as mean ±SD. Different superscript letters (a, b) denote significant differences among groups at the same time point, $p<0.05$. An asterisk (*) indicates a significant effect as determined by two-way repeated-measures ANOVA with Bonferroni post-hoc test ($p<0.05$). DHEA-S (dehydroepiandrosterone sulfate).
